# Prevalence of Neurocognitive Disorders in the Elderly Quechua Population Using the Q-RUDAS

**DOI:** 10.3390/brainsci15121307

**Published:** 2025-12-04

**Authors:** Jonathan Zegarra-Valdivia, Ruth Diana Mamani Quispe, José Chinoapaza Turpo, Carmen Paredes-Manrique, Marco Malaga, Oscar Mamani-Benito, Rosa Montesinos, Nilton Custodio, Giuseppe Tosto

**Affiliations:** 1 Psychology Program, Faculty of Health Sciences, Universidad Cientifica del Sur, Lima 15842, Peru; 2 Escuela Profesional de Psicología, Universidad Peruana Unión, Puno 21000, Peru; ruth.mq@upeu.edu.pe (R.D.M.Q.); jose.ct@upeu.edu.pe (J.C.T.); 3 Facultad de Psicología, Universidad Tecnológica del Perú, Lima 15046, Peru; c27709@utp.edu.pe; 4 Unidad de Investigación, Instituto Peruano de Neurociencias, Lima 15046, Peru; marco.malaga@ucsf.edu (M.M.); rmontesinos@ipn.pe (R.M.); ncustodio@ipn.pe (N.C.); 5 Unidad de Diagnóstico de Deterioro Cognitivo y Prevención de Demencia, Instituto Peruano de Neurociencias, Lima 15046, Peru; 6 Psychology Deparment, Universidad Señor de Sipán, Chiclayo 14001, Peru; oscar.mb@upeu.edu.pe; 7 Servicio de Neurología, Instituto Peruano de Neurociencias, Lima 15046, Peru; 8 Escuela Profesional de Medicina Humana, Universidad Privada San Juan Bautista, Lima 15067, Peru; 9 Tau Institute for Research on Alzheimer’s Disease and the Aging Brain, College of Physicians and Surgeons, Columbia University, New York, NY 10032, USA; gt2260@cumc.columbia.edu; 10 The Gertrude H. Sergievsky Center, College of Physicians and Surgeons, Columbia University, New York, NY 10032, USA; 11 Department of Neurology, College of Physicians and Surgeons, New York Presbyterian Hospital, Columbia University, New York, NY 10032, USA

**Keywords:** cognitive impairment, dementia, quechua population, Q-RUDAS, neurocognitive disorders

## Abstract

Background:The Rowland Universal Dementia Assessment Scale (RUDAS) is a validated cognitive screening tool for illiterate and low-educated individuals, adaptable across languages and cultures. In Peru, we adapted it for Quechua speakers (Q-RUDAS) to assess cognitive status in older adults. Objective: We aimed to estimate the prevalence of neurocognitive disorders—mild cognitive impairment (MCI) and dementia—among Quechua-speaking older adults in one of the most socially vulnerable districts of Peru using the Quechua version of the Rowland Universal Dementia Assessment Scale (Q-RUDAS), a brief cognitive screening tool validated in Peru. Methods: We studied 511 participants from Puno a region in the southern Peruvian Andes (mean age 65.04 ± 6.73 years; 80.4% females), collecting sociodemographic data and Q-RUDAS scores. After excluding 18 individuals with medical conditions that could affect cognitive performance, such as neurological, psychiatric, or cerebrovascular disorders, 493 completed the test. Results: All Q-RUDAS items were well understood, although over 50% of participants struggled with visuospatial construction. The mean Q-RUDAS score was 26.01 ± 2.71. Of the participants, 446 (90.5%) scored within normal ranges (26.67 ± 1.92), 41 (8.3%) were classified as having mild cognitive impairment (MCI) (21.49 ± 1.92), and 6 (1.2%) as having dementia (17.00 ± 2.71) based on established Q-RUDAS cut-offs. Urban participants scored higher. The prevalence of MCI and dementia combined was 9.52%. Conclusions: The Q-RUDAS is a culturally sensitive tool that can support the identification of cognitive impairment in Indigenous populations. Our findings highlight the need for further cross-validation studies to refine diagnostic accuracy in Quechua-speaking populations.

## 1. Introduction

Indigenous populations from Canada, Australia, the United States, Guam, and Brazil have been reported to show a higher prevalence of chronic conditions, including neurocognitive disorders (NCDs), which tend to affect individuals at a younger age due to socioeconomic and health disparities [1].

According to the Diagnostic and Statistical Manual of Mental Disorders, Fifth Edition (DSM-5), neurocognitive disorders (NCDs) encompass conditions characterized by a decline from a previous level of cognitive functioning, classified as mild NCD (commonly referred to as mild cognitive impairment, MCI) and major NCD (or dementia). In MCI, individuals exhibit measurable cognitive deficits in one or more domains but maintain independence in daily activities, whereas dementia involves more pronounced impairments that interfere with autonomy and everyday functioning [2]. The causes of NCDs are multifactorial, including vascular, neurodegenerative, and metabolic factors. In Peru, recent evidence has shown that older age, low educational attainment, hypertension, and hearing loss are major risk factors for MCI and dementia [3]. Similarly, studies in urban-marginalized populations of Lima have identified additional determinants such as depression, low socioeconomic status, and non-Spanish native language use as contributors to increased NCD risk [2]. These findings emphasize the need to consider sociocultural and linguistic diversity when assessing cognitive decline in Peruvian and Indigenous populations.

Studies of Indigenous and rural low-literacy populations report widely varying prevalence rates of dementia, ranging from 0.5% to 26.8% among individuals aged 60 years and older, whereas the prevalence of mild cognitive impairment (MCI) or mild NCD has been reported between 4.4% and 17.7% [4]. For example, among Indigenous Bolivians, the crude prevalence of MCI was 7.7% in the Tsimane population and 9.8% in the Mosetén population, while the crude prevalence of dementia was 1.2% in the Tsimane and 0.6% in the Mosetén among the lowest reported worldwide [5]. Some Indigenous Bolivian and Peruvian populations show surprisingly low rates of cardiovascular risk factors (CVRFs) compared with other Hispanic/Latino groups [6]. A survey found that, although the prevalence of obesity in rural Puno is approximately 75%, only 12% of participants had hypertension and 3% had diabetes [7]. Genetic factors, the lack of refined sugar in their diet, or higher levels of physical activity might contribute to the reduced risk of cardiovascular and metabolic diseases, which in turn may lower the risk of dementia. The low prevalence of the APOE-ε4 allele might also be relevant, as apolipoproteins play an important role in lipid metabolism; the APOE-ε4 allele is associated with higher LDL-cholesterol levels and increased risks for cardiovascular conditions [8]. Explanations for this observation—often referred to as the “Hispanic Paradox” may relate to environmental factors (e.g., stronger social support and culture-specific resilience) and differences in genetic background. For example, in Aymara and Quechua populations, global Native American ancestry (NA) has shown a protective effect against Alzheimer’s disease and related disorders among APOE-ε4 non-carriers [9].

In Peru, 4,390,088 individuals speak an Indigenous language as their native tongue, representing 16.3% of the national population. Among them, 3,375,682 people (13.9%) are Quechua speakers [10]. However, there is currently no reliable estimate of the prevalence of mild cognitive impairment (MCI) or dementia in these communities, primarily due to the challenges of applying standardized diagnostic batteries. The cultural adaptation of neuropsychological instruments that account for the cognitive abilities and daily experiences characteristic of Indigenous life is essential to accurately assess learning, memory, and information-processing skills, and, consequently, to identify pathological cognitive impairment [11].

The process of transcultural adaptation of cognitive screening instruments involves developing versions of assessment tools that are equivalent to the original while being linguistically and culturally adapted to a different context. The Rowland Universal Dementia Assessment Scale (RUDAS) is less affected by culture, language, and education compared with other brief screening instruments for the detection of dementia [12]. Multiple investigations conducted in culturally and linguistically diverse samples from High-Income Countries (HICs) such as Australia and the United Kingdom have reported minimal effects of cultural and language factors on RUDAS performance [13,14]. Although some research has found that limited (<7 years) or no formal education may influence scores, particularly in the visuospatial construction and praxis items, this generally does not affect diagnostic accuracy when the cutoff value is lowered by one or two points in these participants. An advantage of the RUDAS across Low- and Middle-Income Countries (LMICs) and HIC settings appears to be its suitability for dementia screening in populations with limited or no formal education. In Peru, the RUDAS, compared with expert clinical diagnosis, has shown superior discrimination between individuals with mild cognitive impairment (MCI) and dementia than between those with normal cognition and MCI in both urban [15] and rural [16] communities.

Previous community-based research in Peru has used cognitive screening instruments that were not validated for the populations in which they were applied for example, the Mini-Mental State Examination (MMSE), which is not appropriate for individuals with low educational levels. Using such instruments, a dementia prevalence of 6.85% was reported [17]. In contrast, using the RUDAS, mild cognitive impairment (MCI) was identified in 37.3% of Peruvians aged 60 years and older [2]. Moreover, these reports are outdated and given the rapid increase in the elderly population worldwide, the prevalence of dementia may have risen in Peru. Therefore, in this study, we aimed to assess cognitive status and estimate the prevalence of neurocognitive disorders (MCI and dementia) among older adults in their native language, in one of the most socially vulnerable districts of Peru, using a brief cognitive screening tool validated in the country.

## 2. Materials and Methods

### 2.1. Sample and Study Design

This was an observational, descriptive, cross-sectional study. We assessed 511 participants from the Puno region, approached in the Juliaca community through a non-probabilistic, convenience sampling method [18]. We used multiple approaches, including door-to door visits, community outreach, and recruitment through municipal centers, health posts, and local markets.

Inclusion criteria were age 59 years or older; Quechua speakers; no history of untreated psychiatric or neurological disorders; and no sensory difficulties (hearing or visual) that impair evaluation. Participants were required to have normal daily functioning and to provide voluntary participation and sign informed consent.

Exclusion criteria included a history of dementia, psychiatric or neurological disorders, cerebrovascular disease, or intellectual disability.

All participants spoke Quechua as a first or second language. A total of 493 participants were included in the study (81.1% were female), with a mean age of 64.57 ± 6.07 years. We also assessed sociodemographic characteristics, including areas of residence (urban, rural, or mixed) and the number of languages spoken by the participants.

### 2.2. Procedures

Direct and inverse translation of the Rowland Universal Dementia Assessment Scale (RUDAS) was conducted as part of a pilot adaptation study carried out by researchers in the city of Cusco, in southern Peru [19]. Three bilingual Quechua-speaking translators participated in the forward, backward, and review phases of the translation of the Peruvian version of the RUDAS (originally in Spanish). The translated version was reviewed three times by a panel of neurologists, psychologists, and general practitioners fluent in Quechua until a final consensus was reached. The resulting Quechua version of the RUDAS (Q-RUDAS) demonstrated good comprehension and acceptability among native Quechua speakers during pilot testing.

Two bilingual psychologists both native Quechua speakers from the Puno region were trained to administer both versions of the RUDAS (first in Spanish and then in Quechua). They conducted the evaluations following the finalized Quechua adaptation. After each assessment, participants were invited to provide open-ended feedback on the clarity and cultural relevance of the test items.

### 2.3. Instrument Description

The Rowland Universal Dementia Assessment Scale (RUDAS) is a widely used cognitive screening instrument, particularly suitable for individuals with limited or no formal education [20]. It has been translated into several languages and is considered a multicultural tool with high ecological validity and minimal cultural or educational bias. The RUDAS comprises six domains: memory (registration and recall), visuospatial orientation, visuospatial construction, praxis, judgment, and language, with a total possible score of 30 points distributed across these areas.

The RUDAS has been validated in the Peruvian population in both urban and rural settings, showing adequate psychometric properties, including good internal consistency and concurrent validity [14,15]. For illiterate or low-educated individuals, Cronbach’s alpha was 0.65 and Spearman’s correlation 0.79 (*p* < 0.01). The area under the ROC curve (AUC) was 98.0% for differentiating dementia from MCI (cut-off < 19; sensitivity 95%, specificity 97%) and 98.0% for distinguishing MCI from controls (cut-off < 23; sensitivity 89%, specificity 93%) [15]. For individuals with medium to high educational levels, Cronbach’s alpha was 0.68 and Pearson’s correlation 0.79 (*p* < 0.01), with an AUC of 89.0% to discriminate dementia from MCI (cut-off < 21) and 99.0% to differentiate MCI from controls (cut-off < 24) [14].

**Figure 1:**
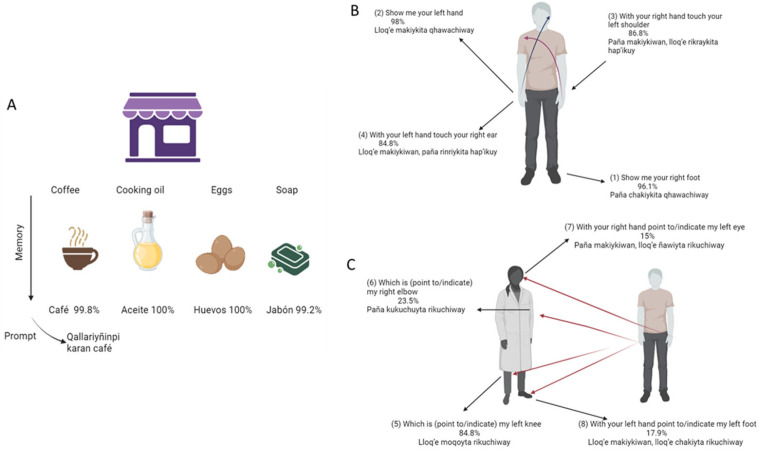
(**A**–**C**) Participants understanding and realization accuracy (%) of Q-RUDAS items (memory and visuo-spatial orientation) Notes: Initial items of Q-RUDAS and their respective translation. Percentage of accuracy is shown on each item.

**Figure 2:**
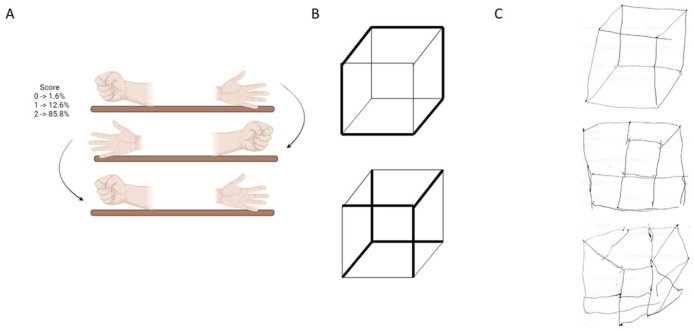
Participants understanding and realization accuracy (%) of Q-RUDAS items (Praxias and Visuo-spatial construction) Notes: (**A**): Praxias movements and their percentage of understanding and realization. (**B**): Cube example in the visuo-spatial construction domain. (**C**): Examples of cube drawing. Upper, good cube drawing. Middle and below, altered drawing.

**Figure 3:**
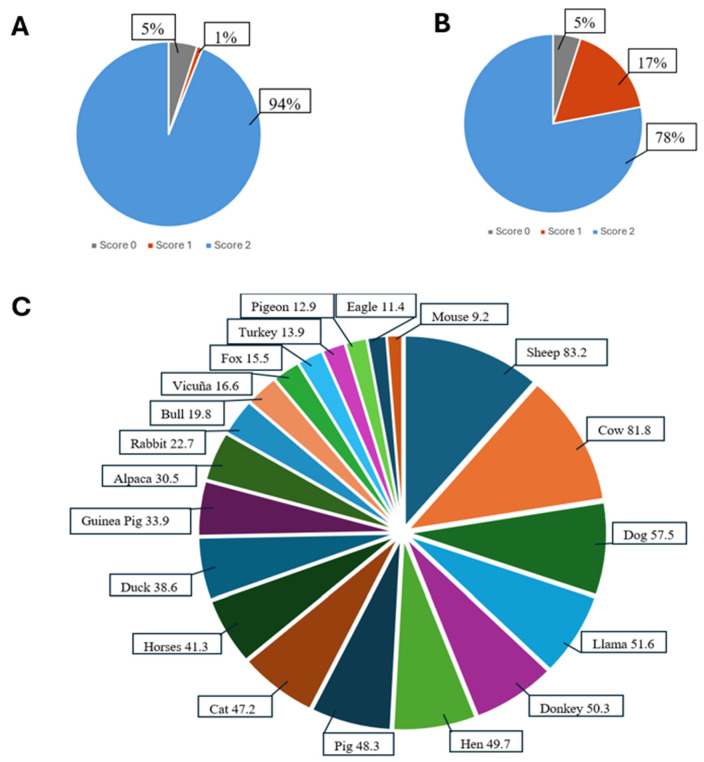
Participants understanding and realization accuracy (%) of Q-RUDAS items (memory and orientation) Notes: (**A**): Scores on the realization of judgment task 1. (**B**): Scores on the realization of judgment task 2. (**C**): First 20 most frequent animals in the language task.

In our adaptation, we used a post-test questionnaire to evaluate participants’ understanding and performance on each test item (see Figure 1, Figure 2 and Figure 3). The average administration time for the RUDAS was approximately 15–20 min.

### 2.4. Ethical Considerations

The institutional research and local ethics review boards approved the study protocol, and all procedures were conducted in accordance with the latest revision of the Declaration of Helsinki. Ethical approval was granted by the Peruvian Union University Ethics Committee (approval number 2023-CEUP-eU-042). All participants were informed about the study’s objectives and procedures.

A total of 459 participants provided written informed consent, while 52 illiterate participants gave verbal consent, which was confirmed through a statement signed by their legal guardian(s). Participation was voluntary and anonymous, and a bilingual psychologist invited participants to take part in the study in their native language. Patients or members of the public were not involved in the design or implementation of the research.

### 2.5. Covariables

For further analysis, we examined the effects of several covariates on cognitive performance. Age was measured in years, and sex was categorized as female or male. Education was recorded in years of formal schooling and further classified into three categories: Non-educated, Low-educated (fewer than 6 years), and Educated (6 years or more). Occupation was categorized as Unemployed, Farmer, Independent (including merchants, vendors, and other trades), Technical, or Professional. The participants’ place of origin was classified as rural, urban, or mixed. Marital status was recorded as single, cohabitant (partners living together), married, divorced, or widowed. Finally, language proficiency was assessed by identifying participants as monolingual (Quechua only) or bilingual (Spanish and Quechua).

### 2.6. Statistical Analysis

To ensure data quality and reliability, we first conducted preliminary analyses. Descriptive statistics (frequencies, percentages, measures of central tendency, and dispersion) were computed. Distributional assumptions were examined with the Kolmogorov–Smirnov test and homogeneity of variances with Levene’s test. Given the distribution of the data, nonparametric tests were used where appropriate: χ^2^ tests for categorical variables, Mann–Whitney U tests for two-group comparisons (e.g., by sex), and Kruskal–Wallis H tests for three-group comparisons (e.g., by educational level). For nonparametric analyses, Monte Carlo estimation with 10,000 repetitions was employed to obtain accurate *p*-value approximations. Where relevant (e.g., comparison of a mean against a theoretical reference), one-sample *t* tests were reported separately.

To evaluate test homogeneity and validity criteria, item–test correlations (cognitive domains vs. total Q-RUDAS score) were estimated using Pearson’s r. Internal consistency was assessed with Cronbach’s alpha, along with its confidence limits.

Subsequently, we fitted an ANCOVA to examine associations between predictors and cognitive performance (total Q-RUDAS score), adjusting for age, sex, years of education, and place of origin, and testing relevant interactions. Preliminary regression models and a final ANCOVA model were used to confirm predictors; bias-corrected bootstrapped 95% confidence intervals (10,000 resamples) were computed to address deviations from normality. All analyses were conducted in SPSS v22.0. Figures were produced with BioRender 3.2. Statistical significance was set at *p* < 0.05 (*), *p* < 0.01 (**), and *p* < 0.001 (***).

## 3. Results

### 3.1. Validity of the Q-RUDAS Score in Feasibility and Reliability

Most of the participants identified as Quechuas (473, 95.9%) and only 20 participants identified as Aymara (4.1%) natives. Nonetheless, they also spoke Quechua. For all participants, we verified their understanding and correct performance on each Q-RUDAS item across cognitive domains. Regarding the Memory domain (Figure 1A) Almost all participants understood the cue words in Quechua for coffee/*café* (99.8%), cooking oil/*aceite*(100%), eggs/*huevos*(100%) and soap/*jabón*(99.2%) in the Quechua language. Similarly, the visuospatial orientation domain showed very good comprehension and performance in the first five items (Figure 1B), whereas the more challenging items are shown in Figure 1C.

Figure 2A shows the praxis movement that participants were asked to perform during the evaluation, placing both hands on the table after observing or imitating the examiner. Figure 2B presents the cube that participants were instructed to draw, and Figure 2C provides examples of participants’ drawing performance.

Figure 3A shows the percentage of participants who understood and answered the first task in the judgment domain. Figure 3B presents the second task from the same domain. Finally, Figure 3C displays the frequency of animals mentioned by participants within 60 s during the language domain evaluation.

Table 1 presents the Pearson correlations obtained to assess the test’s homogeneity and determine its criterion validity (item-test correlations). As can be observed, some domains of the test showed significant correlations, ranging from 0.146 (Judgment and language) to 0.278 (Visuo-construction and Praxis). In addition, the correlations with the total Q-RUDAS score ranged from 0.349 (Judgment) to 0.743 (Memory), indicating moderate and significant relationships between each domain and the total test score. Other domains also showed high correlations with the total Q-RUDAS score, such as Visuo-construction (0.567) and Praxis (0.413).

Furthermore, Table 2 displays the internal consistency (Cronbach’s alpha) calculated using 16 items (considering only the first five corrected V-S orientation items) and 19 items (including items 6 to 8 in the V-S orientation domain). For V-S orientation, the correct score requires only 5 points. Nonetheless, we calculated internal consistency for both configurations, and both showed acceptable internal consistency (16 items: α = 0.726; 95% confidence interval: 0.689–0.760; 19 items: α = 0.634; 95% confidence interval: 0.586–0.680).

### 3.2. Sociodemographic Characteristics, Associations, and Prevalence of Mild Cognitive Impairment and Dementia

Table 3 presents the sociodemographic characteristics of the participants (*n* = 493; 93 males and 400 females) and their Q-RUDAS scores according to sex and educational level. Men were significantly older than women, U = 13,827, *p* < 0.001 (M = 66.68, SD = 6.52 vs. M = 64.09, SD = 5.87). Significant sex differences were also found for occupation, χ^2^(4, *n* = 493) = 12.80, *p* = 0.016; educational level, U = 14,202, *p* < 0.001; and place of origin, χ^2^(2, *n* = 493) = 8.24, *p* = 0.016. Regarding the Q-RUDAS subscales, a significant difference between males and females was observed only in visuospatial construction, U = 13,827, *p* < 0.001. No other sociodemographic variables or cognitive subscales showed significant sex-related differences.

Regarding educational level, significant differences were observed in age, H(2) = 37.34, *p* < 0.001; sex, χ^2^(2, *n* = 493) = 13.19, *p* = 0.002; and marital status, χ^2^(4, *n* = 493) = 25.74, *p* = 0.002. Additional group differences emerged for spoken language, H(2) = 23.48, *p* < 0.001, and place of origin, χ^2^(2, *n* = 493) = 45.53, *p* < 0.001. In the Q-RUDAS subscales, significant effects of educational level were found for visuospatial orientation, H(2) = 12.18, *p* = 0.003; praxis, H(2) = 16.74, *p* < 0.001; and visuospatial construction, H(2) = 71.42, *p* < 0.001. The total Q-RUDAS score also differed significantly across educational groups, H(2) = 33.19, *p* < 0.001.

Table 4 presents the analysis of differences in Q-RUDAS scores according to language and place of origin. Participants were divided into monolinguals (Quechua only; mean score = 78.87 ± 5.52, *n* = 23) and bilinguals (Quechua and Spanish; mean score = 63.87 ± 5.17, *n* = 470). The comparison revealed significant differences between both groups (U = 625, *p* < 0.001), with monolingual participants obtaining higher Q-RUDAS total scores.

Differences were also observed in marital status (χ^2^ = 13.403, *p* = 0.024), years of education (χ^2^ = 2505.5, *p* < 0.001), and place of origin (rural, urban, or mixed; χ^2^ = 8.067, *p* = 0.015). Regarding Q-RUDAS subscales, significant differences were found in praxis (U = 3750, *p* < 0.001) and visuospatial construction (U = 3976.5, *p* = 0.023). The total Q-RUDAS score also differed significantly between groups (U = 3709.5, *p* = 0.009).

In Table 5, an Analysis of Covariance (ANCOVA) was conducted to determine the effect of age, sex, education, and origin on Q-RUDAS scores. The results revealed that the covariate sex was only significant for the construction score (visuospatial and visuo-constructive domain), with a minimal effect (F = 5.83, *p* < 0.017 *; partial η^2^ = 0.026). Age had a greater impact on the praxis score (F = 2.836, *p* < 0.001 *; partial η^2^ = 0.277), memory (F = 2.017, *p* < 0.003 *; partial η^2^ = 0.214), and language (F = 3.389, *p* < 0.001 *; partial η^2^ = 0.314). Additionally, age influenced the total score (F = 2.622, *p* < 0.001 *; partial η^2^ = 0.261). Education significantly affected praxis (F = 2.039, *p* < 0.019 *; partial η^2^ = 0.11) and visuospatial construction (F = 3.438, *p* < 0.001 *; partial η^2^ = 0.172) scores. No significant differences were found for the origin covariate.

The analysis also included interaction terms to examine the combined effects of covariates. The interaction between sex and age affected visuospatial orientation (F = 2.645, *p* < 0.003 *; partial η^2^ = 0.129), while the interaction between sex and education also influenced visuospatial orientation (F = 2.113, *p* < 0.03 *; partial η^2^ = 0.081). The interaction between age and education showed significant effects on praxis (F = 2.302, *p* < 0.001 *; partial η^2^ = 0.515), visuospatial construction (F = 1.403, *p* < 0.021 *; partial η^2^ = 0.392), critical judgment (F = 1.415, *p* < 0.019 *; partial η^2^ = 0.394), and language (F = 1.395, *p* < 0.023 *; partial η^2^ = 0.391). Similarly, the interaction between age and origin influenced praxis (F = 3.229, *p* < 0.001 *; partial η^2^ = 0.222), language (F = 2.515, *p* < 0.001 *; partial η^2^ = 0.182), and the total score (F = 1.717, *p* < 0.035 *; partial η^2^ = 0.132). The interaction between education and origin affected language (F = 1.663, *p* < 0.033 *; partial η^2^ = 0.151). Lastly, no significant differences were observed for the interaction between sex and origin.

Table 6 displays the initial prevalence analysis of cognitive impairment and dementia according to Q-RUDAS cut-off points in a population with low and moderate education levels in Peru [15]. It was found that 90.5% (*n* = 446) corresponded to normal scores, 8.30% (*n* = 41) were individuals with MCI, and 1.22% (*n* = 6) of the population presented with dementia. In the urban sector, 95.30% (*n* = 163) had normal scores, 4.70% (*n* = 8) were individuals with MCI, and no cases of dementia were found. On the other hand, in the rural sector, 87.50% (*n* = 232) had normal scores, 10.60% (*n* = 28) were individuals with MCI, and 1.90% (*n* = 5) had dementia scores. Additionally, it was observed that 86.5% (*n* = 51) of the mixed population (individuals living in both urban and rural areas) had normal scores, 8.80% (*n* = 5) were individuals with MCI, and 1.80% (*n* = 1) presented with dementia.

According to gender, 94.6% (*n* = 88) of males and 89.5% (*n* = 358) of females had normal scores; 5.4% (*n* = 5) of males and 9.0% (*n* = 36) of females had MCI, and 1.5% (*n* = 6) of females presented with dementia. Regarding language, participants who reported being monolingual showed that 73.9% (*n* = 17) had normal scores, 21.7% (*n* = 5) had MCI, and 4.3% (*n* = 1) had dementia. Among bilingual participants, 91.3% (*n* = 429) had normal scores, 7.7% (*n* = 36) had MCI, and 1.1% (*n* = 5) had dementia. Considering the level of education, among non-educated participants, 73.1% (*n* = 38) had normal scores, 21.2% (*n* = 11) had MCI, and 5.8% (*n* = 3) had dementia. For low-educated participants, 93.1% (*n* = 285) had normal scores, 5.9% (*n* = 18) had MCI, and 1.0% (*n* = 3) had dementia. Finally, among educated participants, 91.1% (*n* = 123) had normal scores, and 8.9% (*n* = 12) had MCI. No dementia cases were found in this group.

## 4. Discussion

To our knowledge, this is the first community-based study using the Rowland Universal Dementia Assessment Scale (RUDAS) among Quechua-speaking individuals with low educational levels (Mean = 5.23; SD = 3.79) living in urban–rural areas of Peru. It is also the first study to evaluate the performance of the RUDAS in estimating the prevalence of mild cognitive impairment (MCI) and dementia in this vulnerable population. Our findings highlight two main points: (1) the Quechua version of the RUDAS (Q-RUDAS) was well understood among native participants, and (2) the estimated prevalence of neurocognitive disorders (NCDs) in this population was approximately 9.52%.

Previously, RUDAS was validated in patients referred to memory clinics, who are typically highly selected, whereas a community sample is far more heterogeneous. In this study, the presence of cognitive symptoms was not a requirement for participation. We found that persons from the city of Juliaca, located in southern Peru, performed significantly worse on the domains of motor praxis and visuospatial construction (more than 50% could not draw the cube, more than 25% failed to draw some parts, and around 20% drew it correctly) on the Q-RUDAS, particularly among people living in rural areas and monolinguals, as evidenced by the effects of age and education according to the analysis of covariance (Table 5). Similar results were observed in our previous study [16], which included two culturally distinct cohorts of illiterate participants from rural communities in the Peruvian jungle (Santa Clotilde and Chuquibambilla). Compared to illiterate participants from the city of Lima, these rural groups performed significantly worse on the same cognitive domains of the Q-RUDAS, suggesting that educational level and certain sociocultural or linguistic factors present in rural environments may influence performance on the Q-RUDAS among illiterate individuals.

Additionally, several studies using the RUDAS have shown that low educational attainment is associated with poor performance in alternating hand movements and cube copying [13,21]. In general, the assessments of praxis such as orofacial movements, fine alternating finger movements, imitation of non-sensical movements, coordinated bimanual tasks, line cancellation, and motor impersistence tend to be more challenging for illiterate individuals compared to highly educated professionals [22,23]. Moreover, illiterate people often struggle with cube drawing and the reproduction of three-dimensional figures [16]. Compared with highly educated individuals, illiterate persons have been shown to experience greater difficulty when copying figures (cube, house, intersecting pentagons, complex Rey–Osterrieth figure), recognizing superimposed figures, interpreting maps, or drawing floor plans of rooms [24].

The internal consistency of the Q-RUDAS (16 items, α = 0.726, 95% Interval confidence: 0.689 to 0.760; 19 items, α = 0.634, 95% Interval confidence: 0.586 to 0.680) is in line with previous findings from Peru about illiterate urbans (Cronbach’s alpha = 0.65) and illiterate rural (Cronbach’s alfa = 0.5 in Chuquibambilla and 0.6 in Santa Clotilde) and investigations in Brazil and Australia ranging from 0.54 [25] to 0.80 [26]. Although the performance on the cognitive tests differs across the diagnostic groups, it is essential to highlight that using culturally adapted tools for functional capacity and mood could expand current findings, as these aspects influence cognitive performance and are mediated by sociocultural factors. With a valid and culturally sensitive tool, the next step is to convene a community-based stakeholder advisory board composed of Indigenous older adults, family caregivers, and primary care providers to assess the actions, actors, context, target, and time in which the RUDAS should be administered in monolingual population. This can potentially enhance Indigenous representation in dementia prevalence studies in lower and middle-income countries while also setting the stage for further validation as a useful screening tool for cognitive decline in regular health assessment for older Indigenous individuals. In other hand, comparing Brazilian Indigenous Cognitive Assessment (BRICA) [27], the Brazilian version of Kimberley Indigenous Cognitive Assessment (KICA), the BRICA showed superior sensitivity (MMSE = 67.9%, mKICA = 57.1%, RUDAS = 60.7%) and specificity (MMSE = 97.6%, mKICA = 99%, and RUDAS 92.3%) for dementia. So, there are other options to consider valid for application in this population, with the KICA recommended for illiterate/low-educated individuals [28]. The Q-RUDAS presents several advantages over other available tools such as the BRICA (Brazilian Indigenous Cognitive Assessment) and the KICA (Kimberley Indigenous Cognitive Assessment). Unlike these instruments, which were developed for specific Indigenous populations in Australia and Brazil, the Q-RUDAS is directly adapted from the Rowland Universal Dementia Assessment Scale and linguistically tailored for Quechua speakers. This adaptation ensures cultural and linguistic equivalence while maintaining the psychometric robustness of the original version. Additionally, the Q-RUDAS requires minimal literacy, can be administered in 15–20 min, and has demonstrated high feasibility and comprehension among participants in rural Andean settings. While BRICA and KICA show good sensitivity and specificity in their respective populations, the Q-RUDAS offers a more practical and validated option for cognitive screening in non-Spanish speaking Andean communities [20].

Our study shows low prevalence rates of NCD (9.52%), including MCI (8.30%) and dementia (1.22%). Meanwhile, a cross-sectional door-to-door study from an urban-marginalized district (Puente Piedra) of the Peruvian capital city, Lima, reported NCD prevalence rates of 25.6% in younger adults and 41.8% in older adults [2], findings that are consistent with other studies conducted among older adults from urban-marginalized communities in Latin America [29] and Ecuador [30], which reported NCD prevalence rates of 34.7% and 43.9%, respectively. Rates observed in this urban and rural Quechua population are closer to the lowest rates reported among rural indigenous Bolivians [5], rural Amazonian populations [31], rural Indian agrarian populations [32], and a Cree native population in Manitoba [33], but differ markedly from the highest age-standardized prevalence rates reported among Australian Aboriginals [34] and Chamorros on Guam [35]. However, differences between our results and previous findings may be related to the use of diverse methodologies across studies. The low prevalence of dementia in aboriginal populations probably occurs due to a physically active lifestyle and low rates of cardiovascular disease, diabetes, and obesity [6,36,37,38], which may protect brain health [39] despite a high burden of parasitic and bacterial infections [40]. Interestingly, when these communities move to urban areas and lose these socio-environmental factors [41], rates of cognitive impairment tend to increase [42]. Moreover, joint and meta-analyses including individuals from six studies across four different populations Mexicans, Mexican Americans, Peruvians, Bolivians, and Caribbean Hispanics found a nominally significant protective effect of the APOE-ε2 allele against the risk of Alzheimer’s disease and related disorders [43], which may help explain the low prevalence of NCD observed in our study.

All the cases of dementia found were in women, and most of them (83.3%) lived in rural areas and were bilingual. Similarly, cases of MCI were more common in rural areas, females, and monolingual participants. However, since this sample included more than 80% women, this may reflect socio-cultural aspects explaining such findings: men are often reluctant to be evaluated and/or are engaged in work activities outside the home. In Latin America and the Caribbean (LAC) countries, brain clocks—neuroimaging models that estimate biological brain age and quantify discrepancies between brain age and chronological age have been used to study sex and regional differences in brain aging. Moguilner et al. [44] reported larger brain age gaps in females in both control and Alzheimer’s disease groups compared with males from eight non LAC countries. Structural socioeconomic inequality, pollution, and health disparities were influential predictors of increased brain age gaps. In this sense, fewer years of education were associated with reduced gray matter volume and lower functional connectivity in key brain regions. Notably, the LAC cohort exhibited lower educational levels than the U.S., and these disparities played a crucial role in shaping geographical differences in gray matter volume and connectivity [45]. These studies contrast with those conducted in the indigenous Bolivian population, where in Tsimane and Mosetén males, certain parietal and occipital structures mediating visuospatial abilities exhibited small but significant increases in regional volume with age [46]. On the other hand, in Latin America, vertical and horizontal inequalities often coexist. For example, people living in rural areas, women, and minority ethnic groups have higher incidences of poverty and extreme poverty. Moreover, women in the region are more likely to have received less formal education and are less likely than men to hold paid jobs, leaving them at greater risk of low or no income and limited pension entitlements in later life, which in turn increases dementia risk among women [47]. Additionally, recent studies have reported that higher levels of inequality are linked to reduced brain volume and disrupted connectivity, particularly in temporo-posterior and cerebellar regions essential for memory and cognitive function. These effects were more pronounced in LAC populations [48].

### Limitations and Strengths

Due to the complexity of conducting a community-based study and resource limitations, it was not possible to perform a complete medical evaluation to determine the specific etiology of dementia or to apply a comprehensive neuropsychological battery. Moreover, we were unable to perform a functional assessment due to the absence of an informant at home or their refusal to participate in the study. Another potential limitation is that we included participants who were functioning normally in their daily lives. This means that individuals with neurological or psychiatric dysfunctions may have been underrepresented. Consequently, those participants who were more limited and possibly had dementia might have been missed.

Additionally, cases were defined solely based on brief cognitive test results; however, this approach follows the design and methodology used in other investigations [2,49] and is consistent with diagnostic approaches that do not require an assessment of the patient’s functionality [50]. Another likely limitation could be the predominantly female sample, meaning that the observed prevalence of dementia may not fully represent what occurs in mixed-gender communities. Nevertheless, studies from Latin America have reported a higher prevalence of dementia among women (8.97%) than men (7.26%) and higher rates among rural residents than urban ones (8.68% vs. 7.71%, respectively). Participants without formal education presented more than double the prevalence of dementia (21.37%) compared to those with at least one year of formal education (9.88%). However, a recent study in Europe found no overall sex or gender difference in dementia risk. The prevalence of modifiable risk factors for dementia differed by sex/gender and socioeconomic status. Dementia risk was higher among those who experienced childhood deprivation and among individuals with lower occupational attainment and wealth. Although differences in the associations between modifiable risk factors and incident dementia were found across subgroups, a potential sex/gender difference in dementia risk related to low cognitive activity was identified, suggesting an increased risk for women compared to men with low cognitive activity [51].

The principal strengths of this study deserve to be highlighted. First, we conducted the cognitive assessment among older adults using a brief screening test (Q-RUDAS) that was previously translated and adapted for the Quechua population and has been validated in Peru across various educational levels [14], including individuals with low education [15] and rural populations [16]. The same tool has also been used in a door to door study in Lima, Peru [15], and is currently being employed in the IMPACT study [52], a global health project focused on the physical and mental health of both patients with dementia and caregivers in four Peruvian cities.

Future research in indigenous communities should include door to door probabilistic sampling, clinical and informant interviews, a comprehensive culturally adapted and validated cognitive battery, neuroimaging, and biomarker assessments to more precisely determine the prevalence of NCD and the influence of risk factors. Improving communication and diagnostic tools could help reduce delayed treatment or misdiagnosis. Providing a standardized screening tool may also increase awareness of cognitive impairment among healthcare professionals. The Q-RUDAS offers a quantitative measure that is easy to interpret and can facilitate communication about cognitive symptoms. It also contributes to a common “language” among healthcare professionals and can support decision-making regarding further evaluation at the primary care level.

This study has some limitations. Its cross-sectional design does not allow for the assessment of cognitive changes over time; thus, longitudinal studies are needed to better understand cognitive decline trajectories in Quechua-speaking populations. In addition, the findings may not be generalizable to other Indigenous groups or Quechua speakers from different regions due to dialectal and cultural differences. The predominance of female participants (around 80%) may also limit the interpretation of gender-specific trends in cognitive impairment.

## 5. Conclusions

This study demonstrates the feasibility and usefulness of the Quechua-adapted Rowland Universal Dementia Assessment Scale (Q-RUDAS) as a brief cognitive screening tool among Indigenous populations with low educational levels. The low prevalence of possible neurocognitive disorders (NCDs) identified in this study highlights the importance of further research using longitudinal and clinically validated approaches. The implementation of culturally and linguistically adapted instruments such as the Q-RUDAS can enhance diagnostic accuracy among non-Spanish speaking Indigenous populations. These findings emphasize the urgent need to integrate culturally sensitive cognitive screening tools into primary healthcare systems to support early detection, timely referral, and the development of public health strategies for dementia prevention and management in vulnerable communities.

## Figures and Tables

**Table 1:** Pearson Correlation for Q-RUDAS Scores (Item-Test Correlation).

		Orientation	Praxis	Visuo-Construction	Judgment	Memory	Language	Q-RUDAS Total Score
Orientation	**R**	1	0.073	0.127 **	0.069	0.084	−0.009	0.352 **
	***p***		0.103	0.005	0.126	0.061	0.842	<0.001
Praxis	**R**	0.073	1	0.201 **	0.278 **	0.086	0.156 **	0.413 **
	***p***	0.103		<0.001	<0.001	0.057	0.001	<0.001
Visuo-Construction	**R**	0.127 **	0.201 **	1	0.008	0.144 **	0.116 **	0.567 **
	***p***	0.005	0		0.852	0.001	0.01	<0.001
Judgment	**R**	0.069	0.278 **	0.008	1	−0.009	0.146 **	0.349 **
	***p***	0.126	0	0.852		0.838	0.001	<0.001
Memory	**R**	0.084	0.086	0.144 **	−0.009	1	0.017	0.743 **
	***p***	0.061	0.057	0.001	0.838		0.706	<0.001
Language	**R**	−0.009	0.156 **	0.116 **	0.146 **	0.017	1	0.308 **
	***p***	0.842	0.001	0.01	0.001	0.706		<0.001
Q-RUDAS Total Score	**R**	0.352 **	0.413 **	0.567 **	0.349 **	0.743 **	0.308 **	1
	***p***	<0.001	<0.001	<0.001	<0.001	<0.001	<0.001	

**Notes:** R: Pearson Correlation; *p*: Statistically significant difference at *p* < 0.01 **.

**Table 2:** Internal Consistency of Q-RUDAS through Cronbach’s Alpha.

No. of Elements	16	19 ^a^
**Cronbach’s Alpha**	0.726	0.634
**Lower limit**	0.689	0.586
**Upper limit**	0.76	0.68
***t*** **value**	3.645	2.735
***gl***	492	491
***p*** **-value**	<0.001	<0.001

**Notes:** ^a^: Includes the last three additional orientation items. *t* value and *gl* correspond to a one-sample Student’s *t*-test performed on the mean total score of the instrument. Cronbach’s Alpha, together with its lower and upper confidence limits, represents the internal consistency reliability of each scale.

**Table 3:** Sociodemographic and Q-RUDAS scores of the participants by sex and educational level.

	Total (*n* = 493)		Males (*n* = 93)		Females (*n* = 400)		U	*p*	NE (*n* = 52)		LE (*n* = 306)		E (*n* = 135)		H	*p*
	Mean	SD	Mean	SD	Mean	SD			Mean	SD	Mean	SD	Mean	SD		
Age	64.57	6.072	66.68	6.516	64.09	5.865	13,287	**0.000 ^b^**	70.08	8.693	64.49	5.743	62.64	4.001	37.343	**0.000 ^c^**
Sex																
Male	93	18.90%	-----		-----		-----		2	3.80%	55	18.00%	36	26.70%	13.191 ^a^	**0.002 ^b^**
Female	400	81.10%	-----		-----		-----		50	96.20%	251	82.00%	99	73.30%		
Single	5	1.00%	2	2.20%	3	0.80%	7.640 ^a^	0.102 ^b^	1	1.90%	1	0.30%	3	2.20%	25.741 ^a^	**0.002 ^b^**
Cohabitant	149	30.20%	25	26.90%	124	31.00%			8	15.40%	107	35.00%	34	25.20%		
Married	252	51.10%	56	60.20%	196	49.00%			27	51.90%	149	48.70%	76	56.30%		
Divorce	49	9.90%	4	4.30%	45	11.30%			5	9.60%	30	9.80%	14	10.40%		
Widower	38	7.70%	6	6.50%	32	8.00%			11	21.20%	19	6.20%	8	5.90%		
Unemployed	38	7.70%	1	1.10%	37	9.30%	12.798 ^a^	**0.016 ^b^**	5	9.60%	20	6.50%	13	9.60%	8.450 ^a^	0.374 ^b^
Farmer	76	15.40%	15	16.10%	61	15.30%			9	17.30%	53	17.30%	14	10.40%		
Independent	364	73.80%	71	76.30%	293	73.30%			38	73.10%	224	73.20%	102	75.60%		
Technical	12	2.40%	4	4.30%	8	2.00%			-----		8	2.60%	4	3.00%		
Professional	5	1.00%	2	2.20%	1	0.30%			-----		1	0.30%	2	1.50%		
Education (years)	5.23	3.794	6.57	3.815	4.92	3.725	14,202	**0.000 ^b^**	-----		3.88	1.836	10.3	2.134	368.339	**0.000 ^c^**
Monolingual	23	4.70%	4	4.30%	19	4.80%	0.034 ^a^	0.556	9	17.30%	13	4.20%	-----		23.481 ^a^	**0.000 ^b^**
Bilingual	470	95.30%	89	95.70%	381	95.30%			43	82.70%	293	95.80%	135	100%		
Urban	171	34.70%	41	44.10%	130	32.50%	8.244 ^a^	**0.016 ^b^**	1	1.90%	104	34.00%	66	48.90%	45.525 ^a^	**0.000 ^b^**
Rural	265	53.80%	48	51.60%	217	54.30%			47	90.40%	168	54.90%	50	37.00%		
Mix	57	11.60%	4	4.30%	53	13.30%			4	7.70%	34	11.10%	19	14.10%		
V-S Orientation	4.75	0.558	4.76	0.519	4.74	0.567	18,397	0.823 ^b^	4.48	0.804	4.76	0.54	4.81	0.449	12.176	**0.003 ^c^**
Praxis	1.84	0.407	1.88	0.357	1.83	0.418	17,805	0.310 ^b^	1.63	0.561	1.86	0.395	1.89	0.338	16.74	**0.000 ^c^**
V-S Construction	1.97	0.993	2.42	0.838	1.87	0.998	12,904.5	**0.000 ^b^**	1.13	0.841	1.9	0.985	2.47	0.78	71.415	**0.000 ^c^**
Judgment	3.6	0.675	3.55	0.7	3.61	0.67	17,725	0.400 ^b^	3.4	0.823	3.59	0.672	3.69	0.604	5.434	0.065 ^c^
Memory	6.09	1.668	6.04	1.615	6.09	1.682	18,063.5	0.648 ^b^	5.69	2.147	6.12	1.586	6.15	1.632	1.423	0.492 ^c^
Language	7.89	0.485	7.92	0.337	7.88	0.513	18,202	0.458 ^b^	7.85	0.724	7.88	0.478	7.91	0.376	0.373	0.836 ^c^
Total score	26.13	2.551	26.58	2.066	26.03	2.643	16,698	0.122 ^b^	24.19	3.236	26.12	2.376	26.91	2.234	33.187	**0.000 ^c^**

**Notes:** U = Mann–Whitney U test; H = Kruskal–Wallis H test; NE = non-educated; LE = low-educated; E = educated; SD = standard deviation. Superscripts: ^a^ χ^2^ test; ^b^ *p*-values estimated by bootstrapping (10,000 resamples); ^c^ *p*-values estimated by Monte Carlo simulation (10,000 samples).

**Table 4:** Sociodemographic and Q-RUDAS scores of the participants by languages and origin.

	Monolingual (*n* = 23)		Bilingual (*n* = 470)		U	*p*	Urban (*n* = 171)		Rural (*n* = 265)		Mix (*n* = 57)		H	*p*
	Mean	SD	Mean	SD			Mean	SD	Mean	SD	Mean	SD		
Age	**78.87**	5.521	63.87	5.171	625	**0.000 ^b^**	63.29	4.296	65.77	7.088	62.88	4.036	10.319	**0.005 ^c^**
Male	**4**	17.40%	89	18.90%	0.034 ^a^	0.853	41	24.00%	48	18.10%	4	7.00%	8.244 ^a^	**0.014 ^b^**
Female	**19**	82.60%	381	81.10%			130	76.00%	217	81.90%	53	93.00%		
Single	**-----**		5	1.10%	13.403 ^a^	**0.024 ^b^**	2	1.20%	2	0.80%	1	1.80%	15.016 ^a^	0.056 ^b^
Cohabitant	**6**	26.10%	143	30.40%			55	32.20%	71	26.80%	23	40.40%		
Married	**11**	47.80%	241	51.30%			88	51.50%	145	54.70%	19	33.30%		
Divorce			49	10.40%			15	8.80%	23	8.70%	11	19.30%		
Widower	**6**	26.10%	32	6.80%			11	6.40%	24	9.10%	3	5.30%		
Unemployed	**4**	17.40%	34	7.20%	6.430 ^a^	0.197 ^b^	9	5.30%	21	7.90%	8	14.00%	19.517 ^a^	**0.013 ^b^**
Farmer	**6**	26.10%	70	14.90%			22	12.90%	50	18.90%	4	7.00%		
Independent	**13**	56.50%	351	74.70%			130	76.00%	189	71.30%	45	78.90%		
Technical	-----		12	2.60%			9	5.30%	3	1.10%	-----			
Professional	-----		3	0.60%			1	0.60%	2	0.80%	-----			
Education (years)	**2.09**	2.466	5.38	3.782	2502.5	**0.000 ^b^**	6.65	3.358	4.13	3.707	6.07	3.854	56.507	**0.000 ^c^**
Monolingual	-----		-----		-----		4	2.30%	19	7.20%	-----		8.607 ^a^	0.015 ^b^
Bilingual	-----		-----		-----		167	97.70%	246	92.80%	57	100		
Urban	**4**	17.40%	167	35.50%	8.607 ^a^	**0.015 ^b^**	-----		-----		-----		-----	
Rural	**19**	82.60%	246	52.30%			-----		-----		-----		-----	
Mix	**0**	-----	57	12.10%			-----		-----		-----		-----	
V-S Orientation	**4.52**	0.898	4.76	0.535	4784.5	0.194 ^b^	4.82	0.469	4.71	0.611	4.7	0.533	4.718	0.092 ^c^
Praxis	**1.52**	0.593	1.86	0.39	3750	**0.000 ^b^**	1.88	0.363	1.8	0.451	1.91	0.285	5.006	0.080 ^c^
V-S Construction	**1.48**	1.123	2	0.981	3976.5	**0.023 ^b^**	2.27	0.881	1.82	1	1.81	1.076	23.695	**0.000 ^c^**
Judgment	**3.35**	0.832	3.61	0.666	4510	0.099 ^b^	3.56	0.678	3.62	0.687	3.61	0.62	1.672	0.441 ^c^
Memory	**5.83**	2.081	6.1	1.647	5086.5	0.622 ^b^	6.25	1.641	6.02	1.674	5.89	1.708	2.655	0.267 ^c^
Language	**7.78**	1.043	7.89	0.442	5266	0.721 ^b^	7.89	0.377	7.87	0.563	7.93	0.371	1.298	0.535 ^c^
Total Score	**24.48**	3.273	26.21	2.487	3709.5	**0.009 ^b^**	26.67	2.153	25.84	2.755	25.86	2.445	9.238	**0.010 ^c^**

**Notes:** U = Mann–Whitney U test; H = Kruskal–Wallis H test; NE = non-educated; LE = low-educated; SD = standard deviation. Superscripts: ^a^ χ^2^ test; ^b^ *p*-values estimated by bootstrapping (10,000 resamples); ^c^ *p*-values estimated by Monte Carlo simulation (10,000 samples).

**Table 5:** Sociodemographic influence on Q-RUDAS by ANCOVA analysis.

		Model	Intersection	Sex	Age	Education	Origin	Sex * Age	Sex * Education	Sex * Origin	Age * Education	Age * Origin	Education * Origin
**Orientation ^a^**	**M^2^**	0.353	2062.276	0.049	0.534	0.265	0.178	0.681	0.544	0.071	0.312	0.312	0.203
	**F**	1.372	8004.677	0.191	2.072	1.03	0.69	2.645	2.113	0.277	1.209	1.212	0.787
	***p***	**0.008**	**<0.001**	0.663	0.002	0.424	0.503	**0.003**	**0.03**	0.599	0.127	0.25	0.745
	**η^2^**	0.639	0.974	0.001	0.218	0.059	0.006	0.129	0.081	0.001	0.358	0.097	0.078
**Praxis ^a^**	**M^2^**	0.219	299.106	0.012	0.279	0.2	0.042	0.116	0.082	0.008	0.226	0.317	0.151
	**F**	2.225	3045.355	0.127	2.836	2.039	0.425	1.182	0.836	0.081	2.302	3.229	1.538
	***p***	**<0.001**	**<0.001**	0.722	**<0.001**	**0.019**	0.654	0.298	0.584	0.777	**<0.001**	**<0.01**	0.061
	**η^2^**	0.741	0.934	0.001	0.277	0.11	0.004	0.062	0.034	0.000	0.515	0.222	0.141
**Construcction ^a^**	**M^2^**	1.248	364.958	3.771	0.767	2.224	1.207	0.474	0.405	0.045	0.907	0.511	0.631
	**F**	1.929	564.317	5.83	1.186	3.438	1.867	0.733	0.626	0.069	1.403	0.79	0.976
	***p***	**<0.001**	**<0.001**	**0.017**	0.244	**<0.001**	0.157	0.719	0.774	0.793	**0.021**	0.718	0.498
	**η^2^**	0.713	0.724	0.026	0.138	0.172	0.017	0.039	0.026	0.000	0.392	0.065	0.095
**Judgment ^a^**	**M^2^**	0.511	1131.145	0.838	0.585	0.588	0.315	0.5	0.411	0.308	0.545	0.509	0.592
	**F**	1.326	2933.792	2.174	1.518	1.525	0.817	1.298	1.065	0.798	1.415	1.32	1.536
	***p***	**0.015**	**<0.001**	0.142	0.051	0.11	0.443	0.221	0.39	0.373	**0.019**	0.172	0.061
	**η^2^**	0.631	0.932	0.01	0.17	0.084	0.008	0.068	0.043	0.004	0.394	0.104	0.141
**Memory ^a^**	**M^2^**	2.872	2980.08	0.037	5.373	2.129	1.303	1.651	1.089	3.739	2.697	2.862	2.18
	**F**	1.078	1118.499	0.014	2.017	0.799	0.489	0.62	0.409	1.403	1.012	1.074	0.818
	***p***	0.282	**<0.001**	0.907	**0.003**	0.661	0.614	0.824	0.93	0.237	0.463	0.379	0.707
	**η^2^**	0.581	0.839	0.000	0.214	0.046	0.005	0.033	0.017	0.006	0.318	0.087	0.08
**Language ^a^**	**M^2^**	0.263	5772.26	0.042	0.676	0.207	0.36	0.051	0.079	0.082	0.278	0.502	0.332
	**F**	1.315	28,918.944	0.211	3.389	1.039	1.802	0.257	0.396	0.412	1.395	2.515	1.663
	***p***	**0.017**	**<0.001**	0.647	**<0.001**	0.415	0.167	0.995	0.936	0.521	**0.023**	**0.001**	**0.033**
	**η^2^**	0.629	0.993	0.001	0.314	0.059	0.016	0.014	0.016	0.002	0.391	0.182	0.151
**Total Score ^a^**	**M^2^**	7.724	60,520.351	0.498	12.962	4.731	7.357	3.446	0.919	4.701	5.088	8.489	6.808
	**F**	1.562	12,241.456	0.101	2.622	0.957	1.488	0.697	0.186	0.951	1.029	1.717	1.377
	***p***	**<0.001**	**<0.001**	0.751	**<0.001**	0.495	0.228	0.754	0.995	0.331	0.425	**0.035**	0.124
	**η^2^**	0.668	0.983	0.000	0.261	0.055	0.014	0.037	0.008	0.004	0.322	0.132	0.128

**Notes:** a: bootstrapping of 10,000 repetitions for Q-RUDAS total score and its domains; M^2^: Quadratic mean; F: F test; *p*: Statistically significant difference at *p* < 0.05 *; η^2^: Partial eta squared.

**Table 6:** Prevalence approximation using the Q-RUDAS Scores.

	All (*n* = 493)		Urban (*n* = 171)		Rural (*n* = 265)		Mix (*n* = 57)	
	*n*	%	*n*	%	*n*	%	*n*	%
Control	446	90.50%	163	95.30%	232	87.50%	51	89.50%
MCI	41	8.30%	8	4.70%	28	10.60%	5	8.80%
Dementia	6	1.22%	-----		5	1.90%	1	1.80%
	**Males (** ***n*** **= 93)**		**Females (** ***n*** **= 400)**		**Monolingual (** ***n*** **= 23)**		**Bilingual (** ***n*** **= 470)**	
	***n***	**%**	***n***	**%**	***n***	**%**	***n***	**%**
Control	88	94.60%	358	89.50%	17	73.90%	429	91.30%
MCI	5	5.40%	36	9.00%	5	21.70%	36	7.70%
Dementia	-----		6	1.50%	1	4.30%	5	1.10%
	**Non educated (** ***n*** **= 52)**		**Low educated (** ***n*** **= 306)**		**Educated (** ***n*** **= 135)**			
	***n***	**%**	***n***	**%**	***n***	**%**		
Control	38	73.1	285	93.1	123	91.1		
MCI	11	21.2	18	5.9	12	8.9		
Dementia	3	5.8	3	1	-----			

## Data Availability

The original contributions presented in this study are included in the article. Further inquiries can be directed to the corresponding author.

## Abbreviations

The following abbreviations are used in this manuscript:

AD	Alzheimer’s disease
ANCOVA	Analysis of Covariance
APOE	Apolipoprotein E
CI	Confidence Interval
CVRF	Cardiovascular Risk Factors
HICs	High-Income Countries
LMICs	Low- and Middle-Income Countries
MCI	Mild Cognitive Impairment
MMSE	Mini-Mental State Examination
NCD	Neurocognitive Disorder
Q-RUDAS	Quechua version of the Rowland Universal Dementia Assessment Scale
ROC	Receiver Operating Characteristic
RUDAS	Rowland Universal Dementia Assessment Scale
SPSS	Statistical Package for the Social Sciences
V-S	Visuospatial
